# Biological Activity and Phytochemical Study of *Scutellaria platystegia*

**Published:** 2015

**Authors:** Seyedeh Neda Madani mousavi, Abbas Delazar, Hossein Nazemiyeh, Laleh Khodaie

**Affiliations:** a *Drug Applied Research Center, Tabriz University of Medical Sciences, Tabriz, Iran. *; b *School of Pharmacy, Tabriz University of Medical Sciences, Tabriz, Iran.*

**Keywords:** *Scutellaria platystegia*, Antimalaria, Antioxidant, DPPH, Labiatae

## Abstract

This study aimed to determine biological activity and phytochemical study of *Scutellaria platystegia* (family Labiatae). Methanolic (MeOH) extract of aerial parts of *S. platystegia* and SPE fractions of methanolic extract (specially 20% and 40% methanolic fractions), growing in East-Azarbaijan province of Iran were found to have radical scavenging activity by DPPH (2, 2-diphenyl -1- pycryl hydrazyl) assay. Dichloromethane (DCM) extract of this plant exhibited animalarial activity by cell free method providing IC50 at 1.1876 mg/mL. Crude extracts did not exhibit any toxicity assessed by brine shrimp lethality assay.

Phytochemical study of methanolic extract by using reverse phase HPLC method and NMR instrument for isolation and identification of pure compounds respectively, yielded 2-(4- hydroxy phenyl) ethyl-O-β-D- glucopyranoside from 10% and apigenin 7-O-glucoside, verbascoside and martynoside from 40% SPE fraction. Occurance of verbascoside and martynoside as biochemical markers appeared to be widespread in this genus.

Antioxidant and antimalarial activity of MeOH and DCM extracts, respectively, as well as no general toxicity of them could provide a basis for further *in-vitro* and *in-vivo* studies and clinical trials to develop new therapeutical alternatives.

## Introduction

*Scutellaria* which belongs to Labiatae family, spread throughout the world, with approximately 350 currently recognized species (1-2). Plants of this genus have been traditionally used in China, Korea, and Japan as an agent for activating blood circulation, inducing diuresis and reducing oedema. Some other applications of these plants in folk medicine are due to their anti-inflammatory, antiviral, sedative and antioxidant effects (3). Modern pharmacologic researches on crude extracts and isolated compounds of the plants of this genus, confirmed multiple biological activities, including anticonvulsant (2, 4), prolyl oligopeptidase inhibitory, hepatoprotective (3, 5), memory improvement (6-7) effects. In some *in-vitro* methods, phytochemicals of some species of *Scutellaria*, exhibited potent cytotoxic effects on some of the human tumour cell lines (8-10). Flavonoids (3, 11-12) and neoclerodan diterpenoids(1, 13-14) as well as iridoids (3, 15), phenyl alcohol glycosides and alkaloids (3, 16) have been isolated from several species of *Scutellaria*. Due to Remarkable and diverse biological activities of other species of *scutellaria* genus, this study aimed to evaluate biological activity and identify chemical composition of this plant. To our knowledge there have been no reports on biological activities and chemical composition of *S. platystegia*.

## Experimental

*Plant material*

Aerial parts of *Scutellaria platystegia* (family Labiatae) were gathered from Yam region of East Azarbaijan province of Iran in June 2009. A voucher specimen (Tbz-Fph-724) for this collection has been deposited in the Herbarium of pharmacy faculty, Tabriz University of Medical Sciences, Tabriz, Iran. 100 g of the air dried and grounded sample was extracted by the aid of Soxhlet apparatus using hexane, dichloromethane (DCM) and methanol (MeOH) respectively (1 L for each solvent). Obtained extracts were individually concentrated under vacuum in a rotary evaporator (Heidolph, Germany), yielding 1.67 g, 1.50 g and 24.50 g respectively. 

*Fractionation of methanolic extract*

Fractionation of dried methanolic extract (2 g) was carried out using SPE (solid phase extraction) cartridge (Sep- pak ODS C18), eluting with methanol and water mixture (10:90, 20:80, 40:60, 60:40, 80:20 and 100:0). All SPE fractions were dried by rotary evaporator at a temperature not exceeding 50 ºC. In order to increase the available SPE fractions, procedure was repeated 3 times yielding 1.421, 0.279, 0.655, 0.220, 0.162 and 0.190 g of each fraction respectively. 

*DPPH assay*

Antioxidant activity of extracts and different SPE fractions was performed using DPPH assay. The basis of this experiment was Bleaching of purple coloured methanolic solution of 2, 2-diphenyl -1- pycryl hydrazyl (DPPH) (sigma). In order to obtain antioxidant activity, different sample solution series were prepared. 5 mL of each concentration of methanolic extract and SPE fractions were added to 5 mL of 0.004% methanolic solution of DPPH. After 30 min incubation of solutions at room temperature and bleaching of DPPH, absorption of samples was monitored at 517 nm against a blank. Inhibition of DPPH was calculated as RC_50_ that was extrapolated from dose-response curve. Tests were carried out in duplicate (17-18). 

*Antimalaria assay*

The antimalaria potential of extracts was determined using cell free method which was described by Fitch *et al*. (19) with some modifications (20). Different concentrations of the n-hexan, DCM and methanolic extracts ranging from 0-2 mg/mL in 10% DMSO, were incubated in 300 µL of haematin which was freshly dissolved in 0.1 M NaOH, 10 mM oleic acid and 10 µM HCl. Afterwards 500 mM sodium acetate buffer, pH 5, was added to test tubes for adjusting reaction volume to 1000 µL. Positive control was chloroquine diphosphate in this test. The samples were incubated overnight at 37 ºC with regular shaking. After incubation, samples were centrifuged at 14,000 × g, for 10 min, at 21 °C and the hemozoin pellet repeatedly washed with sonication (30 min, at 21 °C; FS100 bath sonicator; Decon Ultrasonics Ltd.) in 2.5% (w/v) SDS in phosphate buffered saline followed by a final wash in 0.1 M sodium bicarbonate, pH 9.0, until the supernatant became clear which usually happens after 3-5 washes. After the final wash, the supernatant was removed and the pellets were re-suspended in 1 mL of 0.1 M NaOH before determining the hemozoin content by measuring the absorbance at 400 nm (Beckmann DU640 spectrophotometer). Results were recorded as IC%, which is inhibition of heme crystallization compared to chloroquine as positive control, using the following formula: IC% = [(AB–AA)/AB] × 100, where AB and AA are absorbance of blank and test samples respectively. Final results which mean inhibition of hemezoin polymerization have been shown as IC50.

*Brine shrimp lethality assay*

This test is proposed as a preliminary and simple assay to study general toxicity of plant extracts. The eggs of *Artemia salina* purchased from Water Life, Middlesex, UK, were hatched in a flask containing 300 mL of artificial sea water aerated by the aid of an air pump. The flasks were kept in a 29-30 ºC water bath and a bright light was left on. Afterwards, nauplii were hatched after 48 h. 1 mg/mL of *n*-hexan, DCM, MeOH were prepared by dissolving them in 5% DMSO. These solutions were serially diluted to obtain 7 concentrations, by the aid of aerated sea water this experiment was carried out for twice. About ten nauplii were transferred in to each test tube. The number of alive nauplii were counted after 24 h. The control test tubes contained 5% DMSO, saline and podophyllotoxin (21-23).

*Isolation of compounds*

**Figure 1:**
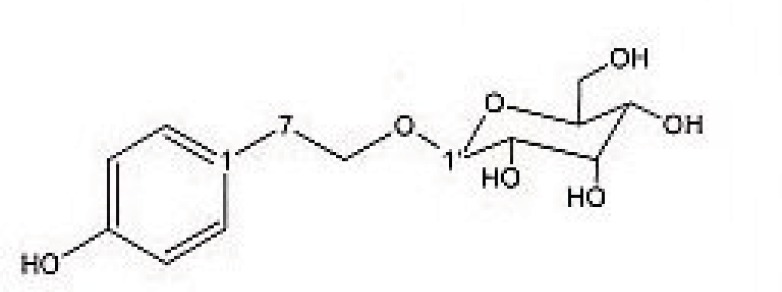
2-(4- hydroxy phenyl) ethyl-O-β-D- glucopyranoside.

**Figure 2:**
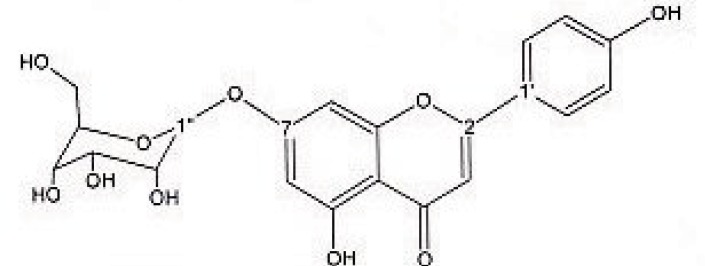
Apigenin 7-O-glucoside.

**Figure 3:**
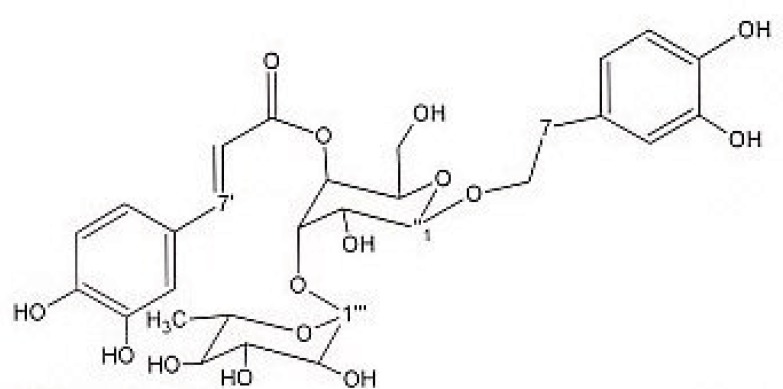
Verbascoside.

**Figure 4:**
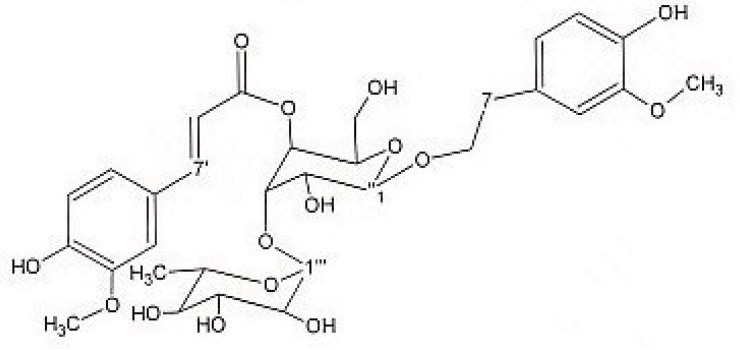
Martynoside.

Preparative reversed phase HPLC with photodiode array detector was used for isolation of phytochemicals from 20 and 40% SPE fractions. Each fraction was analysed repeatedly by preparative reverse phase HPLC (Knauer, preparative pump 1800), equipped with a Reprosil 100 C18 (250 mm length, 20 mm *i.d*, particle size 10 µm, Dr. Maisch, Germany) column. The mobile phase consisted of (A) methanol and (B) water. The following mobile phase program was used over 60 min to isolate glycosylated phenylethanoid (Figure 1) from the 10% SPE fraction: A initially changed to 10% in 15 min. then it changed to 20% in 50 min, maintained there for 10 min. A program over a run time of 50 min was applied for separation of 7-glucoapigenin (Figure 2), verbascoside (Figure 3) and martynoside (Figure 4) from the 40% SPE fraction: 17% A initially changed to 28% in 40 min. Then, it stayed there for 10 min. Photodiode Array Detector (PDA) was used to monitor the chromatogram, and the HPLC separation was carried out at room temperature. The flow rate was 8 mL/min and the injection volume was 1 mL. Structures of compounds were determined by H and C NMR (Bruker-spectrospin at 200MHz) as well as comparison with the literature data of respective compounds.

2-(4- hydroxy phenyl) ethyl-O-β-D- glucopyranoside (Figure 1); pale green solid; ^1^HNMR (200 MHz, D_2_O): Aglycone moiety: δ7.04 (2 H, d, J =7.9 Hz, H2,6), 6.68 (2 H, d, J= 7.9 Hz, H3,5), 3.8-4.1 (2 H , overlapped peak, H8), 2.7 (2 H, t. J= 6.9 Hz, H7), Glucose moiety: δ 4.29 (1 H, d, J = 7.9 Hz, Hl′), 3.2-3.8 (signal patterns unclear due to overlapping, H2′-3′-4′-5′-6′),^13^CNMR (200 MHz, CD3OD): Aglycone moiety: δ 153.88(C4), 130.42(C1), 130.19 (C2,6), 115.28(C3,5), 73.02(C8), 34.22(C7). 

Apigenin 7-O-glucoside (Figure 2); brown solid; ^1^HNMR (200 MHz, CD3OD). Aglycone moiety: δ 7.90 (2H, d, J=8.7, H2',6'), 6.93 (2H, d, J=8.7, H3',5'), 6.85 (1H,d, J=1.8 Hz, H8), 6.63 (1H, S, H3), 6.46 (1H,d, J=1.8 Hz, H6). Glucose moiety: δ 4.65 (1 H, d, J = 7.8 Hz, Hl'), 3.2-3.8 (signal patterns unclear due to overlapping, H2'', H 3'', H 4'', H5''and H6″).

Verbascoside (Figure 3): brown solid; ^1^HNMR (200 MHz, CD3OD): aglycone moiety: δ 7.6 (1H, d, J = 15.8 Hz, H7′), 7.06(1H, d, J=1.82Hz, H2′), 6.98(1H, dd, J =8.1,1.8Hz, H6′), 6.78(1H, d, J=8.1Hz , H5′), 6.69(1H, d, J=1.8Hz, H2), 6.64(1H, d, J=8.1Hz, H5), 6.58(1H, dd, J =8.1,1.8Hz, H6), 6.25 (1H, d, J = 15.8 Hz, H8′), 3.8-4.1 (2H, overlapped peak, H8), 2.79 (2H, t, J = 8 Hz, H7), glucose moiety: δ 4.37 (lH, d, J=7.8Hz, Hl″), 3.2-3.8 (signal patterns unclear due to overlapping, H2″-3″-4″-5″-6″), rhamnose moiety: δ 5.19 (lH, d, J= 1.5 Hz, Hl′′′), 3.2-3.8 (signal patterns unclear due to overlapping, H2′′′-3′′′ -4′′′ -5′′′), 1.085 (3H, d, J=6.0 Hz, H6′′′). ^13^CNMR (200 MHz, CD3OD) Aglycone moiety: δ 166.8(C9′), 148.4(C4′), 146.6(C7′), 145.4(C3′), ), 143.2(C3), 130(C1), 126.2(C1′), 121.8(C6′), 119.8(C6), 115.7(C2), 115.7(C5′), 114.86(C5), 114.7(C4), 113.7 (C2′), 113.2 (C8′), 72.3(C8), 35.1(C7), Glucose moiety: δ 101.6(C1″), 80.2(C3″), 74.8(C2″), 74.6(C5″), 69(C4″), 60.9(C6″), Rhamnose moiety: 102.77(C1′′′), 72.3(C4′′′), 70.9(C2′′′), 70.6(C3′′′), 69.1(C5′′′), 17(C6′′′). Data were in agreement with the published data (24). 

Martynoside (Figure 4); dark solid; ^1^HNMR (200 MHz, CD3OD): aglycone moiety: δ 7.63 (1H, d, J = 15.8 Hz, H7′), 7.16(1H, d, J=1.8Hz, H2′), 7.03(1H, dd, J =8.0,1.8Hz, H6′), 6.79(1H, d, J=8.0Hz, H5), 6.79(1H, d, J=8Hz , H5′), 6.69(1H, d, J=1.8Hz, H2), 6.67(1H, dd, J =8.0,1.8Hz, H6), 6.40 (1H, d, J = 15.8 Hz, H8′), 3.8-4.1 (2H, overlapped peak, H8), 3.87 (3H, S, 3′-OMe), 3.75 (3H, S, 3-OMe), 2.8 (2H, t, J = 7.3 Hz, H7), glucose moiety: δ 4.34 (lH, d, J=7.8Hz, Hl″), 3.2-3.8 (signal patterns unclear due to over lapping, H-2″, H-3″, H-4″, H-5″, H-6″), Rhamnose moiety: δ 5.18 (lH, d, J= 1.7 Hz, Hl′′′), 3.2-3.8 (signal patterns unclear due to overlapping, H-2′′′, H-3′′′, H-4′′′, H-5′′′), 1.085 (3H, d, J=6.0 Hz, H6′′′). ^13^C NMR (200 MHz, CD3OD); Aglycone moiety: δ 166.85 (C9'), 150.81 (C4'), 149.41 (C3'), 148.03 (C7'), 147.98 (C3), 147.37(C4), 132.93 (C1), 127.71 (C1'), 124.46 (C6'), 122.94 (C6), 117.19 (C5), 116.66 (C5'), 115.19 (C8'), 112.95 (C2), 111.91 (C2'), 102.79 (C1'''), 101.58 (C1''), 81.65 (C3''), 76.24 (C2''), 76.04 (C5''), 73.87 (C4'''), 72.42 (C2'''), 72.35 (C8), 72.20 (C3'''), 70.74 (C4''), 70.31 (C5'''), 62.49 (C6''), 53.49 (O-CH3), 53.39 (O-CH3), 35.12 (C7), 17.03 (C6'''). Data were in agreement with the published data (25). 

## Results

DPPH assay was performed to determine Radical scavenging activity of extracts and SPE fractions of this plant. As it can be seen in Table 1, methanolic extract showed better antioxidant activity than other crude extracts. Table 2 demonstrated that among SPE fractions, 20% and 40% hydroalcoholic fractions were more potent antioxidants. Results of antimalarial activity of this plant, which was determined by cell free method, exhibited in Table 3. DCM crude extract, providing IC50 at 1.1876 mg/mL showed potential of antimalarial activity.

**Table 1:** Free radical scavenging activity of the extracts of *S.* *platystegia* by DPPH assay.

**Extract**	***n*** **-hexan**	**DCM**	**MeOH**	**Quercetin**
**RC50 (mg/m)**	1.4283	0.7482	0.0342	0.0039

**Table 2:** Free radical scavenging activity of SPE methanolic fractions of S. *platystegia* by DPPH assay.

**Fraction**	**10%**	**20%**	**40%**	**60%**	**80%**	**100%**	**Quercetin**
**RC50 (mg/mL)**	0.0677	0.0085	0.0144	0.0314	0.2479	0.7321	0.0039

**Table 3:** Antimalarial activity of the extracts of *S*. *platystegia* by inhibition of Heme biocrystallisation assay

**Extract**	***n*** **-hexan**	**DCM**	**MeOH**	**Chloroquin**
**IC50 (mg/mL)**	-	1.1876	16.9356	0.043

Observation of radical scavenging activity from methanolic extract and SPE fractions encouraged us to study this plant phytochemically. Reverse-phase prep-HPLC analysis of SPE fractions of methanolic extract of aerial parts of *S. platystegia* (Labiatae) yielded 2-(4- hydroxy phenyl) ethyl-O-β-D- glucopyranoside from 10% ,one flavonoid (Apigenin 7-O-glucoside) and 2 phenyl ethanoid glycosides (verbascoside and martynoside) from 40% SPE fractions. In fact compounds 1-4 have been identified previously from other species of this genus (26-34), whereas this is the first report of these phytochemicals from *S. platystegia.* Distribution of compounds 1-4 has been demonstrated in Table 4. 

**Table 4:** Distribution of compounds 1-4 within genus *Scutellaria*

	compounds			
*Scutellaria spp.*	1	2	3	4
*S.baicalensis*	+	N.R.*	+	+
*S.baicalensis Georgi*	+	N.R.	N.R.	N.R.
*S. barbata*	N.R.	+	N.R.	N.R.
*S. immaculate*	N.R.	+	N.R.	N.R.
*S. pontifica*	N.R.	+	+	+
*S. ramosissima*	N.R.	+	+	+
*S. salviifolia*	N.R.	N.R.	+	+
*S. orientalis sub. pinnatifida*	N.R.	N.R.	+	+
*S. lateriflora*	N.R.	N.R.	+	+
*S. albida*	N.R.	N.R.	+	+
*S. * *salviifolia*	N.R.	N.R.	+	+
*S. galericulata*	N.R.	N.R.	+	+

* N.R.: not reported

## Discussion

Recently, phytochemicals as bioactive components of plant extracts has received considerable attention. Antioxidants, the agents against oxidative stress-mediated disorders, with free radical scavenging activity, can prevent damages caused by various disorders (35-38). Antioxidant capacity of phenolic compounds isolated from plants (39-42) and Correlation of phenol content and antioxidant activity has been shown in different studies as well (43-44). According to some evidences, flavonoids and phenylethanoids as plant derived polyphenolic compounds act as free radical acceptors, and potent antioxidants (45-49). Plants of *Scutellaria* genus, used in traditional medicine for thousands years, with variety of confirmed pharmacological effects in modern researches, has been shown free radical scavenging and antioxidant activities due to existence of different phenolic compounds such as flavonoids and phenylethanoids (50-57). Results of this study demonstrated that verbascoside, martynoside and apigenin as antioxidant compounds (45, 58-62), which existed in methanolic extract and isolated from 40% fraction were responsible for good radical scavenging activity of this methanolic fraction (0.0342 mg/mL). Further investigations will reveal other phytochemicals responsible for radical scavenging activity of 20% and 60% fractions. Identification of apigenin, martynoside and verbascoside as anti- inflammatory and antioxidant constituents from methanolic extract, is in agreement with traditional usage of this plant as an anti- inflammatory agent (63-66) and confirm its use in folk medicine.

Malaria, a malignancy with worldwide spread, results in loss of lives each year. Resistance of deadly forms of malaria parasites to anti-malarial drugs highlights needs for new antimalarial drugs (67). Anti- fever herbal plants used in traditional medicine might contain some antimalarial phytochemicals which could lead to development of new drugs for treatment of this mortal malignancy (68). Diverse nature of Iran possesses medicinal plants which could be alternative choice for treatment of malaria. In previous works, traditionally used febrifuge plants to treat fever as a symptom of malaria, have been selected for antimalarial studies (69-71). Since several species of *Scutellaria* have been used as febrifuge in folk medicine, extracts of *S. platystegia* were subjected to *in-vitro* antimalarial test (57, 72-73) and interestingly DCM extract of this plant demonstrated antimalarial activity. Further investigations are needed to identify and purify compounds responsible for antimalarial activity of this plant. 

Brine shrimp lethality assay, a suitable, simple, rapid procedure with low cost (74-75), was chosen to determine general toxicity of plant extracts. None of the plant extracts exhibited any toxicity at highest test concentrations (1 mg/mL). 

Biological activity of methanolic extract conducted us to phytochemical study of this plant. 2-(4- hydroxy phenyl) ethyl-O-β-D- glucopyranoside (from 10% SPE fraction), Apigenin-7- O- glucoside, verbascoside and martynoside (from 40% SPE fraction) were isolated and identified by UV and NMR analysis. All spectroscopic data were in agreement with respective published data. Identified components have been reported from other species of *Scutellaria* genus, while distribution of verbascoside and martynoside appears to be widespread (Table 4). So, it is concluded that these two phytochemicals could be determined as chemical biomarkers in this genus. To our knowledge, this is the first report on the antioxidant and antimalarial activity as well as occurrence of compounds 1-4 within this species. 

## Conclusion

It can be concluded that antioxidant and antimalarial activity of MeOH and DCM extracts, respectively, as well as no general toxicity of them, could provide a basis for further *in-vitro* and *in-vivo* studies and clinical trials to develop new therapeutical alternatives. More over the results of present study show that it is worth to do further phytochemical studies on Iranian *Scutellaria* species and to isolate compounds responsible for antioxidant and antimalarial activities. 

## References

[1] Dai SJ, Qu GW, Yu QY, Zhang DW, Li GS (2010). New neo-clerodane diterpenoids from Scutellaria barbata with cytotoxic activities. Fitoterapia.

[2] Zhang Z, Lian XY, Li S, Stringer JL (2009). Characterization of chemical ingredients and anticonvulsant activity of American skullcap (Scutellaria lateriflora). Phytomed.

[3] Shang X, He X, Li M, Zhang R, Fan P, Zhang Q (2010). The genus Scutellaria an ethnopharmacological and phytochemical review. J. Ethnopharmacol.

[4] Park HG, Yoon SY, Choi JY, Lee GS, Choi JH, Shin CY (2007). Anticonvulsant effect of wogonin isolated from Scutellaria baicalensis. Eur. J. Pharmacol.

[5] De BJG, Quiney B, Walter PB, Thomas C, Hodgson K, Murch SJ (2005). Protection against aflatoxin-B1-induced liver mutagenesis by Scutellaria baicalensis. Mutat. Res.

[6] Shang Y, Cheng J, Qi J, Miao H (2005). Scutellaria flavonoid reduced memory dysfunction and neuronal injury caused by permanent global ischemia in rats. Pharmacol. Biochem. Behav.

[7] Heo H, Shin Y, Cho W, Choi Y, Kim H, Kwon YK (2009). Memory improvement in ibotenic acid induced model rats by extracts of Scutellaria baicalensis. J. Ethnopharmacol.

[8] Cha YY, Lee EO, Lee HJ, Park YD, Ko SG, Kim DH (2004). Methylene chloride fraction of Scutellaria barbata induces apoptosis in human U937 leukemia cells via the mitochondrial signaling pathway. Clin. Chim. Acta.

[9] Ozmen A, Madlener S, Bauer S, Krasteva S, Vonach C, Giessrigl B (2010). In-vitro anti-leukemic activity of the ethno-pharmacological plant Scutellaria orientalis ssp carica endemic to western Turkey.. Phytomed.

[10] Kumagai T, Muller CI, Desmond JC, Imai Y, Heber D, Koeffler HP (2007). Scutellaria baicalensis, a herbal medicine: anti-proliferative and apoptotic activity against acute lymphocytic leukemia, lymphoma and myeloma cell lines. Leuk. Res.

[11] Liu G, Ma J, Chen Y, Tian Q, Shen Y, Wang X (2009). Investigation of flavonoid profile of Scutellaria bacalensis Georgi by high performance liquid chromatography with diode array detection and electrospray ion trap mass spectrometry. J. Chromatogr. A.

[12] Sato Y, Suzaki S, Nishikawa T, Kihara M, Shibata H, Higuti T (2000). Phytochemical flavones isolated from Scutellaria barbata and antibacterial activity against methicillin-resistant Staphylococcus aureus. J. Ethnopharmacol.

[13] Miyaichi Y, Kizu H, Yamaguchi Y, Tomimori T (1994). Studies on the constituents of Scutellaria species XV. On the diterpenoid constituents of the leaves of Scutellaria alpina L.. Yakugaku Zasshi.

[14] Ezer N, Akcos Y, Rodrguez B (1998). Neo-clerodane diterpenoids from Scutellaria orientalis subsp sintenisii.. Phytochem.

[15] Gousiadou C, Karioti A, Heilmann J, Skaltsa H (2007). Iridoids from Scutellaria albida ssp albida.. Phytochem.

[16] Dai SJ, Liang DD, Ren Y, Liu K, Shen L (2008). New neo-clerodane diterpenoid alkaloids from Scutellaria barbata with cytotoxic activities. Chem. Pharm. Bull.

[17] Moein S, Moein M, Khoshnoud MJ, Kalanteri T (2012). In-vitro antioxidant properties evaluation of 10 Iranian medicinal plants by different methods. Iran. Red. Crescent Med. J.

[18] Kicel A, Wolbis M (2013). Phenolic content and DPPH radical scavenging activity of the flowers and leaves of Trifolium repens. Nat. Prod. Commun.

[19] Fitch CD (1998). Involvement of heme in the antimalarial action of chloroquine. Trans. Am. Clin. Climatol. Assoc.

[20] Tripathi AK, Gupta A, Garg SK, Tekwani BL (2001). In-vitro beta-hematin formation assays with plasma of mice infected with Plasmodium yoelii and other parasite preparations: comparative inhibition with quinoline and endoperoxide antimalarials. Life Sci.

[21] Meyer B, Ferrigni N, Putnam J, Jacobsen L, Nichols Dj, McLaughlin J (2007). Brine shrimp: a convenient general bioassay for active plant constituents. Planta Medica.

[22] Carballo JL, Hernández-Inda ZL, Pérez P, García-Grávalos MD (2002). A comparison between two brine shrimp assays to detect in-vitro cytotoxicity in marine natural products. BMC Biotechnol.

[23] Hasan MS, Ahmed MI, Mondal S, Uddin SJ, Masud M, Sadhu S (2006). Antioxidant, antinociceptive activity and general toxicity study of Dendrophthoe falcata and isolation of quercitrin as the major component. Int. J. Orient. Pharm. Exp. Med.

[24] Olivier D, Shikanga E, Combrinck S, Krause R, Regnier T, Dlamini T (2010). Phenylethanoid glycosides from Lippia javanica. S. Afr. J. Bot.

[25] Khodaie L, Delazar A, Lotfipour F, Nazemiyeh H, Asnaashari S, Moghadam SB (2012). Phytochemistry and bioactivity of Pedicularis sibthorpii growing in Iran. Rev. Bras. Farmacogn.

[26] Liu YX, Liu ZG, Su L, Yang RP, Hao DF, PeiYH (2009). Chemical constituents from Scutellaria baicalensis Georgi. Chinese J. Med. Chem.

[27] Sato Y, Suzaki S, Nishikawa T, Kihara M, Shibata H, Higuti T (2000). Phytochemical flavones isolated from Scutellaria barbata and antibacterial activity against methicillin-resistant Staphylococcus aureus. J. Ethnopharmacol.

[28] Çaliş I, Ersöz T, Saracoglu I, Sticher O (1993). Scalbidoside and albidoside, two iridoid glycosides from Scutellaria albida subsp. Colchica. Phytochem.

[29] Yuldashev M (2001). Flavonoids of the aerial part of Scutellaria immaculate. Chem. Nat. Compd.

[30] Simmonds MS (2006). The search for plant-derived compounds with antifeedant activity. Adv. Phytomed.

[31] Mamadalieva NZ, Herrmann F, El‐Readi MZ, Tahrani A, Hamoud R, Egamberdieva DR (2011). Flavonoids in Scutellaria immaculata and S ramosissima (Lamiaceae) and their biological activity. J. Pharm. Pharmacol.

[32] Saracoglu I, Inoue M, Calis I, Ogihara Y (1995). Studies on constituents with cytotoxic and cytostatic activity of two Turkish medicinal plants Phlomis armeniaca and Scutellaria salviifolia. Biol. Pharm. Bull.

[33] Çaliş İ, Saracoğlu İ, Başaran AA, Sticher O (1993). Two phenethyl alcohol glycosides from Scutellaria orientalis subsp. Pinnatifida. Phytochem.

[34] Zhang Z, Lian XY, Li S, Stringer JL (2009). Characterization of chemical ingredients and anticonvulsant activity of American skullcap (Scutellaria lateriflora). Phytomed.

[35] Ratnam DV, Ankola D, Bhardwaj V, Sahana DK, Kumar M (2006). Role of antioxidants in prophylaxis and therapy: A pharmaceutical perspective. J. Control Release.

[36] Diplock AT (1991). Antioxidant nutrients and disease prevention: an overview. Am. J. Clin. Nutrit.

[37] Padayatty SJ, Katz A, Wang Y, Eck P, Kwon O, Lee JH (2003). Vitamin C as an antioxidant: evaluation of its role in disease prevention. J. Am. Coll. Nutr.

[38] Zafra‐Stone S, Yasmin T, Bagchi M, Chatterjee A, Vinson JA, Bagchi D (2007). Berry anthocyanins as novel antioxidants in human health and disease prevention. Mol. Nutr. food Res.

[39] Soobrattee MA, Neergheen VS, Luximon-Ramma A, Aruoma OI, Bahorun T (2005). Phenolics as potential antioxidant therapeutic agents: mechanism and actions. Mutat. Res.

[40] 40 (1997). Rice-Evans C, Miller N and Paganga G. Antioxidant properties of phenolic compounds. Trends Plant Sci.

[41] Kähkönen MP, Hopia AI, Vuorela HJ, Rauha JP, Pihlaja K, Kujala TS (1999). Antioxidant activity of plant extracts containing phenolic compounds. J. Agr. Food Chem.

[42] Shahidi F, Janitha P, Wanasundara P (1992). Phenolic antioxidants. Crit. Rev. Food Sci.Nutr.

[43] Lopez-Velez M, Martinez-Martinez F, Valle-Ribes CD (2003). The study of phenolic compounds as natural antioxidants in wine. Crit. Rev. Food Sci.

[44] Turkmen N, Sari F, Velioglu YS (2006). Effects of extraction solvents on concentration and antioxidant activity of black and black mate tea polyphenols determined by ferrous tartrate and Folin–Ciocalteu methods. Food Chem.

[45] Rice-evans CA, Miller NJ, Bolwell PG, Bramley PM, Pridham JB (1995). The relative antioxidant activities of plant-derived polyphenolic flavonoids. Free Radical Res.

[46] Ordonez A, Gomez J, Vattuone M (2006). Antioxidant activities of Sechium edule (Jacq ) Swartz extracts.. Food Chem.

[47] Lee JY, Yoon JW, Kim CT, Lim ST (2004). Antioxidant activity of phenylpropanoid esters isolated and identified from Platycodon grandiflorum A. DC. Phytochem.

[48] Zhang L, Liao CC, Huang HC, Shen YC, Yang LM, Kuo YH (2008). Antioxidant phenylpropanoid glycosides from Smilax bracteata. Phytochem.

[49] Chang CL, Zhang LJ, Chen RY, Kuo LMY, Huang JP, Huang HC (2010). Antioxidant and anti-inflammatory phenylpropanoid derivatives from Calamus quiquesetinervius. J. Nat. Prod.

[50] Huang WH, Lee AR, Yang CH (2006). Antioxidative and anti-inflammatory activities of polyhydroxyflavonoids of Scutellaria baicalensis Georgi. Biosci. Biotech. Bioch.

[51] Cole IB, Cao J, Alan AR, Saxena PK, Murch SJ (2008). Comparisons of Scutellaria baicalensis, Scutellaria lateriflora and Scutellaria racemosa: genome size, antioxidant potential and phytochemistry. Planta Medica.

[52] Gao Z, Huang K, Yang X, Xu H (1999). Free radical scavenging and antioxidant activities of flavonoids extracted from the radix of Scutellaria baicalensis Georgi. BBA- Gen Subjects.

[53] Gabrielska J, Oszmiański J, Zyłka R, Komorowska M (1997). Antioxidant activity of flavones from Scutellaria baicalensis in lecithin liposomes. J. Biosci.

[54] Su YL, Leung LK, Bi YR, Huang Y, Chen ZY (2000). Antioxidant activity of flavonoids isolated from Scutellaria rehderiana. J. Am. Oil Chem. Soc.

[55] Shieh DE, Liu LT, Lin CC (2000). Antioxidant and free radical scavenging effects of baicalein, baicalin and wogonin. Anticancer Res.

[56] Ersoz T, Tasdemir D, Calis I, Ireland C (2002). Phenylethanoid glycosides from Scutellaria galericulata. Turk J. Chem.

[57] Shang X, He X, He X, Li M, Zhang R, Fan P (2010). The genus Scutellaria an ethnopharmacological and phytochemical review. J. Ethnopharmacol.

[58] Benavente-Garcıa O, Castillo J, Lorente J, Ortuno A, Del Rio J (2000). Antioxidant activity of phenolics extracted from Olea europaea L leaves.. Food Chem.

[59] Vertuani S, Beghelli E, Scalambra E, Malisardi G, Copetti S, Toso RD (2011). Activity and stability studies of verbascoside, a novel antioxidant, in dermo-cosmetic and pharmaceutical topical formulations. Molecules.

[60] Wang P, Kang J, Zheng R, Yang Z, Lu J, Gao J (1996). Scavenging effects of phenylpropanoid glycosides from Pedicularis on superoxide anion and hydroxyl radical by the Spin trapping method. Biochem. Pharmacol.

[61] Papoutsi Z, Kassi E, Mitakou S, Aligiannis N, Tsiapara A, Chrousos GP (2006). Acteoside and martynoside exhibit estrogenic/antiestrogenic properties. J. Steroid Biochem.Mol. Biol.

[62] Lu Y, Yeap FL (2001). Antioxidant activities of polyphenols from sage (Salvia officinalis). Food Chem.

[63] Penido C, Costa KA, Futuro DO, Paiva SR, Kaplan MAC, Figueiredo MR (2006). Anti-inflammatory and anti-ulcerogenic properties of Stachytarpheta cayennensis (LC Rich) Vahl. J. Ethnopharmacol.

[64] Kanchanapoom T, Kasai R, Picheansoonthon C, Yamasaki K (2001). Megastigmane, aliphatic alcohol and benzoxazinoid glycosides from Acanthus ebracteatus. Phytochem.

[65] Vo TNN, Phi LT, Lam TP, Lawrence MV, Phung NN, Kim PP (2012). Lignans and Triterpenes from the Root of Pseuderanthemum carruthersii var atropurpureum.. Chem. Pharm. Bull.

[66] Kim HPS, Kun HC, Hyeun WK, Sam S (2004). Anti-inflammatory plant flavonoids and cellular action mechanisms. J. Pharm. Sci.

[67] Azas N, Laurencin N, Delmas F, Di Giorgio C, Gasquet M, Laget M (2002). Synergistic in-vitro antimalarial activity of plant extracts used as traditional herbal remedies in Mali. Parasitol. Res.

[68] Najib Nik A Rahman N, Furuta T, Takane K, Ali Mohd M (1999). Antimalarial activity of extracts of Malaysian medicinal plants. J. Ethnopharmacol.

[69] Ancolio C, Azas N, Mahiou V, Ollivier E, Di Giorgio C, Keita A (2002). Antimalarial activity of extracts and alkaloids isolated from six plants used in traditional medicine in Mali and Sao Tome. Phytother. Res.

[70] Addae-Kyereme J, Croft SL, Kendrick H, Wright CW (2001). Antiplasmodial activities of some Ghanaian plants traditionally used for fever/malaria treatment and of some alkaloids isolated from Pleiocarpa mutica; in-vivo antimalarial activity of pleiocarpine. J. Ethnopharmacol.

[71] Koch A, Tamez P, Pezzuto J, Soejarto D (2005). Evaluation of plants used for antimalarial treatment by the Maasai of Kenya. J. Ethnopharmacol.

[72] Lazari D, Gabrieli C, Papi R, Tsoleridis K, Kyriakidis D (2008). Biological activities of iridoids from Scutellaria rupestris ssp adenotricha.. Planta Medica.

[73] Skaltsa HD, Lazari DM, Kyriazopoulos P, Golegou S, Triantaphyllidis S, Sokovic M (2005). Composition and antimicrobial activity of the essential oils of Scutellaria sieberia Benth. and Scutellaria rupestris Boiss. et Heldr. ssp. adenotricha (Boiss. et Heldr.) Greuter et Burdet from Greece. J. Essent. Oil Res.

[74] Movahhedin N, Barar J, Fathi FA, Barzegari A, Nazemiyeh H (2014). Phytochemistry and biologic activities of Caulerpa peltata native to oman sea. Iran. J. Pharm. Res.

[75] Firuzi O, Miri R, Asadollahi M, Eslami S, jasbi AR (2013). Cytotoxic, antioxidant and antimicrobial activities and phenolic contents of eleven salvia species from iran. Iran. J. Pharm. Res.

